# OVA-PEG-R848 nanocapsules stimulate neonatal conventional and plasmacytoid dendritic cells

**DOI:** 10.3389/fped.2022.966113

**Published:** 2022-09-13

**Authors:** Sebastian Wirsching, Marina Machtakova, Frauke Borgans, Leah Pretsch, Michael Fichter, Maximiliano L. Cacicedo, Héloïse Thérien-Aubin, Katharina Landfester, Stephan Gehring

**Affiliations:** ^1^Children's Hospital, University Medical Center of the Johannes Gutenberg University, Mainz, Germany; ^2^Max Planck Institute for Polymer Research, Mainz, Germany; ^3^Department of Infectious Diseases, University Hospital Frankfurt, Frankfurt, Germany; ^4^Department of Dermatology, University Medical Center of the Johannes Gutenberg University, Mainz, Germany; ^5^Department of Chemistry, Memorial University of Newfoundland, St. John's, NL, Canada

**Keywords:** neonatal dendritic cells, conventional dendritic cells, plasmacytoid dendritic cells, vaccine, R848, nanocapsules

## Abstract

Childhood mortality represents a major issue with 5. 3 million worldwide deaths of children under 5 years of age in 2019. Approximately half of those deaths can be attributed to easily preventable, infectious diseases. Currently approved neonatal vaccines are typically effective only after multiple doses leaving infants especially vulnerable during the first 6 months of life. Survival rates could be improved significantly by developing new and more potent vaccines that are capable of overcoming inherently tolerogenic neonatal immune systems. TLR agonists have garnered a great deal of attention in recent years due to their extensive capacities to activate innate immunity. Herein, the superior capacity of the TLR7/8 agonist, resiquimod (R848), to activate adult and neonatal primary peripheral blood dendritic cells is demonstrated. Moreover, R848 can be conjugated to polyethylene glycol and encapsulated in ovalbumin nanocapsules to efficiently co-deliver antigen and adjuvant *in vitro*. This study is among the first to demonstrate the capacity of encapsulated R848 to activate neonatal dendritic cells. These findings support the potential incorporation of R848 as adjuvant in neonatal vaccines, making them more effective in eliciting a robust immune response.

## Introduction

Easily preventable, infectious diseases accounted for ~50% of the 5.3 million worldwide deaths of children under 5 years of age in 2019. Although childhood mortality has been on a steady decline in recent years, it is projected that, based on current trends, as many as 48.1 million children who are 5 years or younger will die between 2020 and 2030 (1, 2). The development of novel and more potent vaccines that are able to overcome the limitations of the neonatal immune system represents a primary approach to improving the survival rate.

*In utero*, T cells are mainly polarized toward a T_H_2 phenotype in order to prevent fetal allograft rejection (3, 4). Immediately after birth, the immune system is skewed toward a tolerogenic phenotype. This polarization persists through the first few months of life, and is mainly due to a dampened T_H_1-type response leaving infants extremely susceptible to infectious diseases (5). Regulatory T cells, found in cord blood and infant tissues in greater abundance, further contribute to the compromised state of the neonatal immune system (6, 7). The reduced production of pro-inflammatory cytokines, e.g., IL-12p70 and TNF-α, by antigen presenting cells upon Toll-like receptor (TLR) stimulation represents a further contributing factor (8, 9). Additionally, neonatal dendritic cells (DCs) are functionally immature, expressing fewer cell surface MHC II molecules, as well as co-stimulatory receptors: CD40, CD80 and CD86 (9, 10). The administration of Bacille Calmette-Guérin (BCG) vaccine, however, shows that neonatal DCs are fully capable of secreting pro-inflammatory cytokines and mounting a robust T_H_1-type immune response (11, 12).

Conventional and plasmacytoid DCs fulfill different functions but are equally important in presenting antigens and activating T cells. Conventional DCs, in particular the more abundant CD1c-expressing subset, exhibit a broad range of TLRs and are capable of secreting IL-12p70, TNF-α and IL-6 after sensing pathogens (13, 14). CD123-expressing plasmacytoid DCs, on the other hand, are uniquely tailored to respond to viral infections by expressing TLR7 and TLR9, which recognize single-stranded RNA and DNA, respectively, and by producing type I interferons (15).

Apart from BCG, there are only two other vaccines, Hepatitis B (HBV) and polio, that are currently approved for administration to neonates. Of the three, only the HBV vaccine uses aluminum-based (alum) agents as an adjuvant to boost immune responses. Today, the CDC recommends three doses of the HBV vaccine administered during the first 6 months after birth to achieve robust protection (16). A big challenge is the mother-to-infant transmission, which can be prevented effectively by administrating HBV immunoglobulin together with HBV vaccine (17, 18). This, however, can be challenging in low-income countries since infants only infrequently come in contact with the health care system or certain medications, in this case HBV immunoglobulin, are not available at all (19, 20). Therefore, novel and more effective adjuvants, which activate the neonatal immune system early and decrease childhood mortality, are needed to improve vaccines.

TLR agonists have garnered a great deal of attention in recent years due to their extensive capabilities to activate the innate immune system (21). Today, two TLR agonists are approved for use as vaccine adjuvants. Monophosphoryl lipid A (MPLA), a component of the Cervarix vaccine, is a TLR4 agonist; and CpG oligodeoxynucleotides, a component of the Heplisav-B vaccine, is a TLR9 agonist (22, 23).

The study presented herein demonstrates the superior capacity of the TLR7/8 agonist, resiquimod (R848), relative to alum and MPLA, to activate both adult and neonatal primary peripheral blood DCs. Stimulation with R848 increased both activation marker expression and pro-inflammatory cytokine secretion by conventional and plasmacytoid DCs. Adult and neonatal DCs stimulated with R848 coupled to polyethylene glycol (PEG) and encapsulated in ovalbumin nanocapsules (OVA-PEG-R848), herein used as model antigen, exhibited an activated phenotype. This supports the potential use of encapsulated PEG-R848 as a versatile platform to co-deliver a variety of antigens and R848 to DCs.

## Materials and methods

### Synthesis of PEG-R848 and labeling with Rhodamine B

PEG-R848 was prepared as previously described (24). Hydroxyl-PEG-NHS ester (160 mg; Sigma, St. Louis, MO) was dissolved in 4 mL of dry dichloromethane before adding 10 mg R848 (Sigma) and 6 μL trimethylamine. The reaction was stirred overnight. Afterwards, the solvent was evaporated, and water was added to the solid product. The resulting solution was dialyzed against water for 3 days, then freeze-dried. PEG-R848 was labeled with a fluorescent dye by dissolving 50 mg in 4 mL DMSO and adding 2 mg of Rhodamine B isothiocyanate (Sigma). Then, the reaction was stirred overnight, dialyzed against water for 5 days, and lyophilized.

### Synthesis of protein nanocapsules and encapsulation of PEG-R848

Ovalbumin (OVA) and Ovalbumin-polyethylene glycol-R848 (OVA-PEG-R848) nanocapsules (NCs) were synthesized as previously described (24). Briefly, 20 mg of OVA (Sigma) were dissolved in 0.2 mL PBS-buffer (Sigma) and 1 mg of PEG-R848 was added to the aqueous solution. The organic phase was prepared by dissolving 75 mg of polyglycerol-polyricinoleat (PGPR) (Danisco, Copenhagen, Denmark) in 3 mL toluene. Both phases were emulsified with an Ultra-Turrax® (IKA, Staufen im Breisgau, Germany) and homogenized by one cycle through a LV1 microfluidizer (MFIC, Newton, MA). Afterwards, a solution of 25 mg PGPR and 0.1 μL of Toluol-2,4-diisocyanat (TDI) (TCI Chemicals, Tokyo, Japan) in 0.5 mL toluene was added dropwise to the mini-emulsion and then stirred (20 h, RT). The nanocapsules were purified by centrifugation three times (30 min, 1,200 g) followed by redispersion in fresh toluene to remove excess surfactant and unreacted crosslinker. The nanocapsules were then transferred to PBS for subsequent use.

### Nanocapsule characterization

NCs were characterized as described before (24). Shortly, NC size distribution was measured via dynamic light scattering (DLS) at 20°C using a Malvern NanoS90 (Malvern Instruments, UK) device at 90° angle. Zeta potential was measured through dilution of NCs in potassium chloride solution with a Malvern ZetaSizer (Malvern Instruments, UK).

### Encapsulation efficiency and release experiments

Encapsulation efficiency of nanocapsule formulations was measured as described before (24). Shortly, encapsulation efficiency was calculated by measuring the total fluorescence intensity of PEG-Rhodamine (λex = 553 nm and λem = 576 nm) in the aqueous suspension. Afterwards, the samples were centrifuged and the remaining, unencapsulated PEG-Rhodamine in the recovered suspension as well as the PEG-Rhodamine in the redispersed NCs was measured.

Release of payload from NCs was measured as previously described (24). Shortly, the release of the PEG-Rhodamine was analyzed by centrifugation of NCs at 1,770 g for 30 min using Vivaspin® Ultrafiltration tubes (500 μl 1,000 K; Sartorius AG, Germany) and measuring the fluorescence of the filtrate (λex = 553 nm and λem = 576 nm).

### Isolation and culture of primary peripheral blood DCs

Leukocytes were obtained from leukapheresis products collected from healthy, voluntary donors (blood bank of the University Medical Center Mainz) after informed consent. Umbilical cord blood was collected after cesarean section from healthy neonates according to guidelines of the University Medical Center Mainz and after ethical approval of the local ethics committee. Peripheral blood mononuclear cells (PBMCs) were isolated by Biocoll density centrifugation. Afterwards, the purified PBMC fraction was washed twice with Hank's Balanced Salt Solution (HBSS; Sigma). cDC2 and pDCs were isolated in one step using the Blood Dendritic Cell Isolation Kit II (Miltenyi Biotec, Bergisch-Gladbach, Germany) according to the manufacturer's instructions.

### DC stimulation

Primary DCs were cultured and stimulated as described before (24). Shortly, 2 x 10^5^ bulk DCs in serum-free X-VIVO 15 medium were seeded in triplicates into 96-well U-bottom plates. For stimulation with soluble adjuvants, DCs were stimulated with 1 μg/ml MPLA (Sigma), 5 μg/ml Alhydrogel® (2%; Invivogen, San Diego, CA) or 1 μg/ml R848 (Sigma) for 20 h at 37°C and 5% CO_2_.

Cells stimulated with nanocapsules were incubated with OVA-PEG-R848 NCs at an effective R848 concentration of 500 ng/ml. Empty OVA NCs were used in equimolar concentration as control. Cells were stimulated with 500 ng/ml soluble PEG-R848 as a positive control.

### Flow cytometry

The cells were transferred to FACS round-bottom tubes, washed with buffer, then incubated with 10% Privigen® Immunoglobulin solution (CSL Behring, Marburg, Germany) to block Fc receptors. Primary DCs were stained with fluorochrome-conjugated mouse monoclonal antibodies specific for CD83 (FITC), CD80 (PE), CD123 (PE-Cy7), HLA-DR (APC) (all purchased from BD Pharmingen, Franklin Lakes, NJ), CD86 (BV420), CD80 (BV510) (BD Horizon) and CD1c (PerCP-eFluor 710) (eBioscience, San Diego, CA) for 30 min at 4°C. The gating strategy used for the gating of DC populations can be found in Supplementary Figure S1.

### Cytometric bead array

Cell culture supernates, collected after 20 h of incubation, were analyzed using the Human Enhanced Sensitivity Flex system (BD) and the MACSPlex Cytokine assay system (Miltenyi Biotec). The assay was performed with IL-6 and TNF-α beads according to the manufacturer's instructions.

### Statistical analysis

Statistical analyses were performed using GraphPad Prism version 7 (GraphPad Software, San Diego, CA). The data were analyzed for Gaussian distribution using a Shapiro-Wilk normality test. The data were further analyzed using a one-way ANOVA and Tukey's *post hoc* analysis if normality tests were passed; if not, a Kruskal-Wallis test and Dunn's *post hoc* analysis was used. A two-way ANOVA was used to compare the effects of different concentrations of soluble R848 and PEG-R848.

## Results

### R848 activates adult and neonatal primary DCs

**Figure 1:**
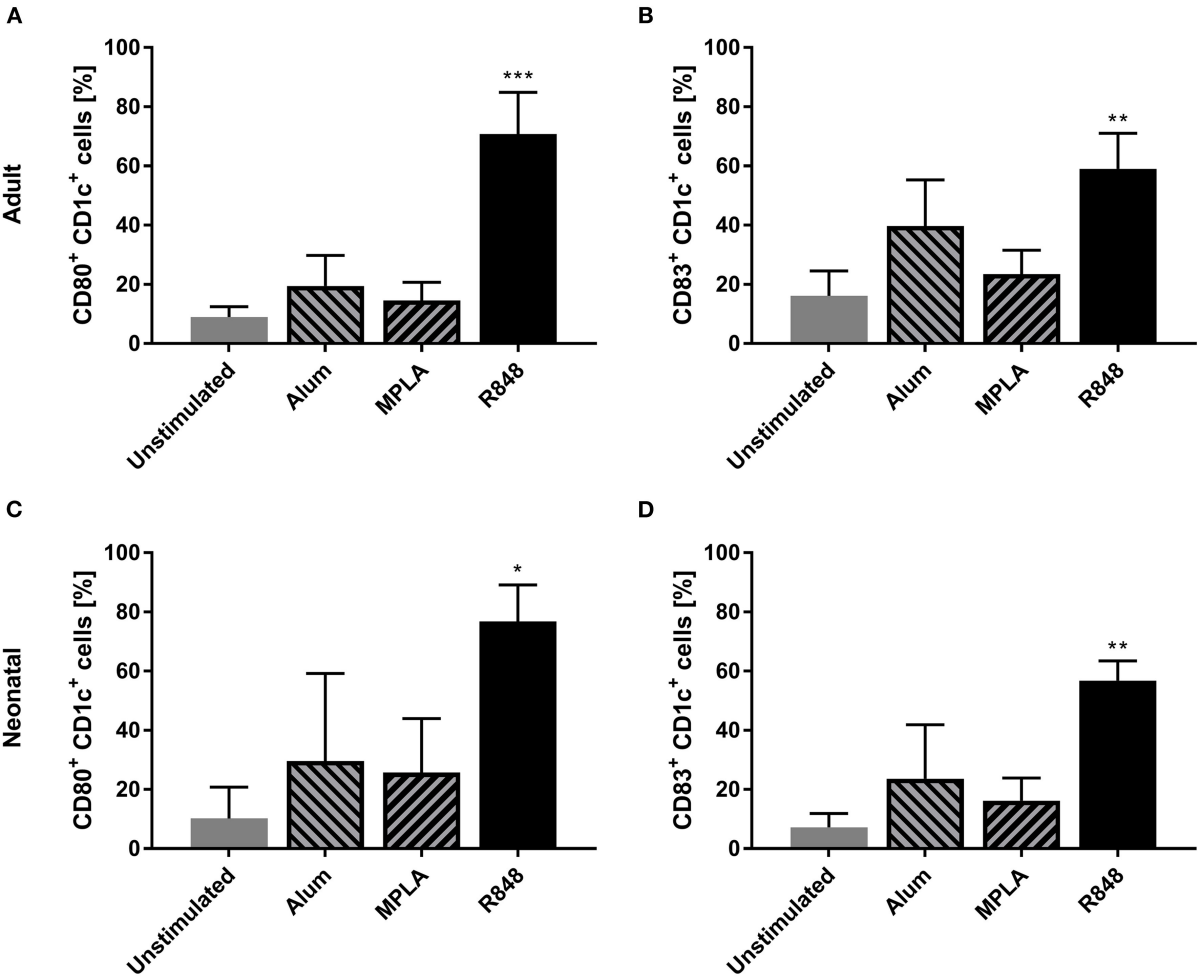
Effect of different adjuvants on CD1c^+^ cDC2 activation. Adult **(A,B)** and neonatal **(C,D)** DCs were incubated with or without alum, MPLA or R848 for 20 h. Flow cytometric analysis of CD80 **(A,C)** and CD83 **(B,D)** expression by CD1c^+^ DCs after stimulation. Data represent the means ± SD (*n* = 3). Significantly greater than unstimulated control: *P < 0.05; **P < 0.001; ***P < 0.005 (one-way ANOVA).

**Figure 2:**
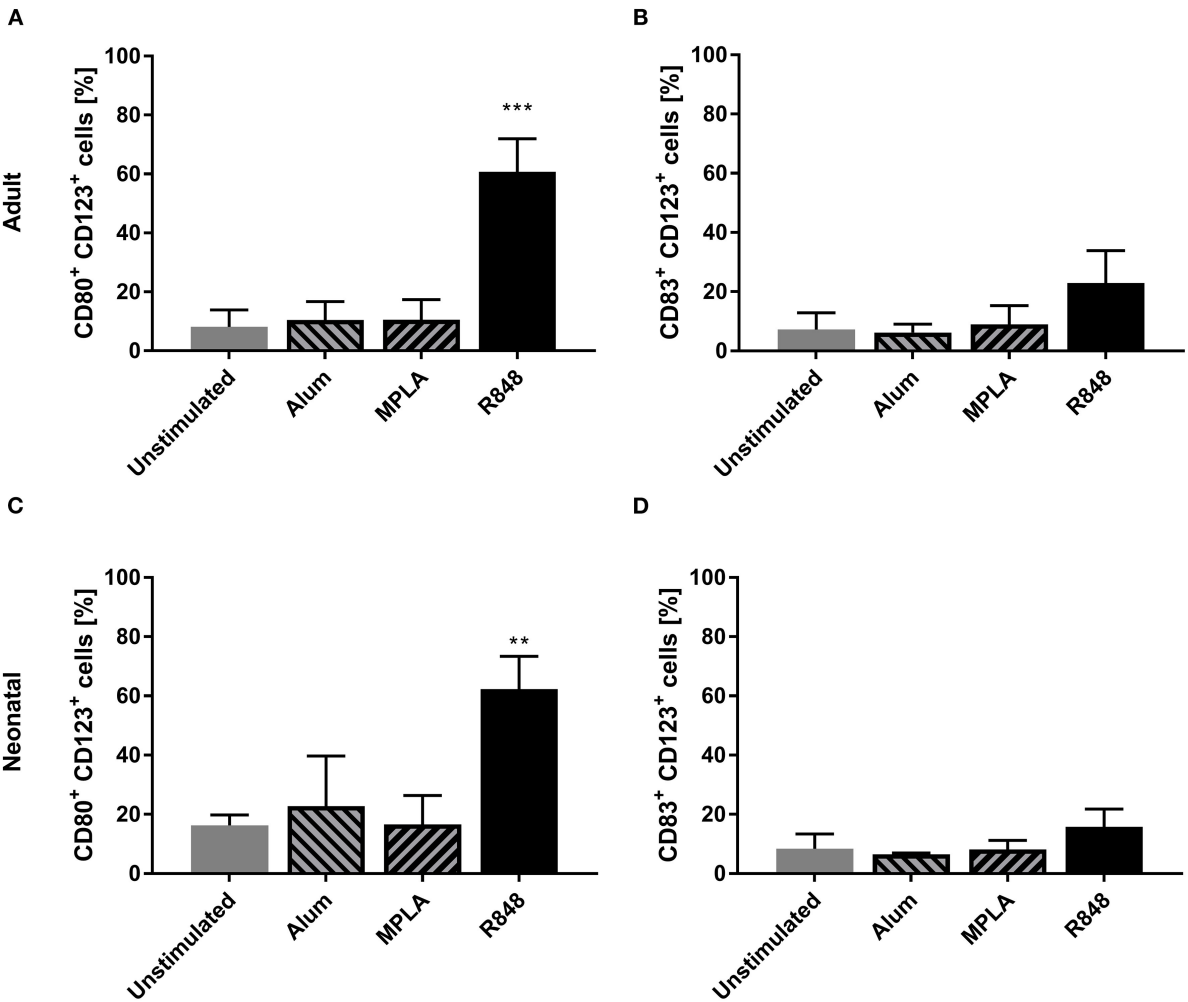
Effect of different adjuvants on CD123^+^ pDC activation. Adult **(A,B)** and neonatal **(C,D)** DCs were incubated with or without alum, MPLA or R848 for 20 h. Flow cytometric analysis of CD80 **(A,C)** and CD83 **(B,D)** expression by CD1c^+^ DCs after stimulation. Data represent the means ± SD (*n* = 3). Significantly greater than unstimulated control: **P < 0.001; ***P < 0.005 (one-way ANOVA).

**Figure 3:**
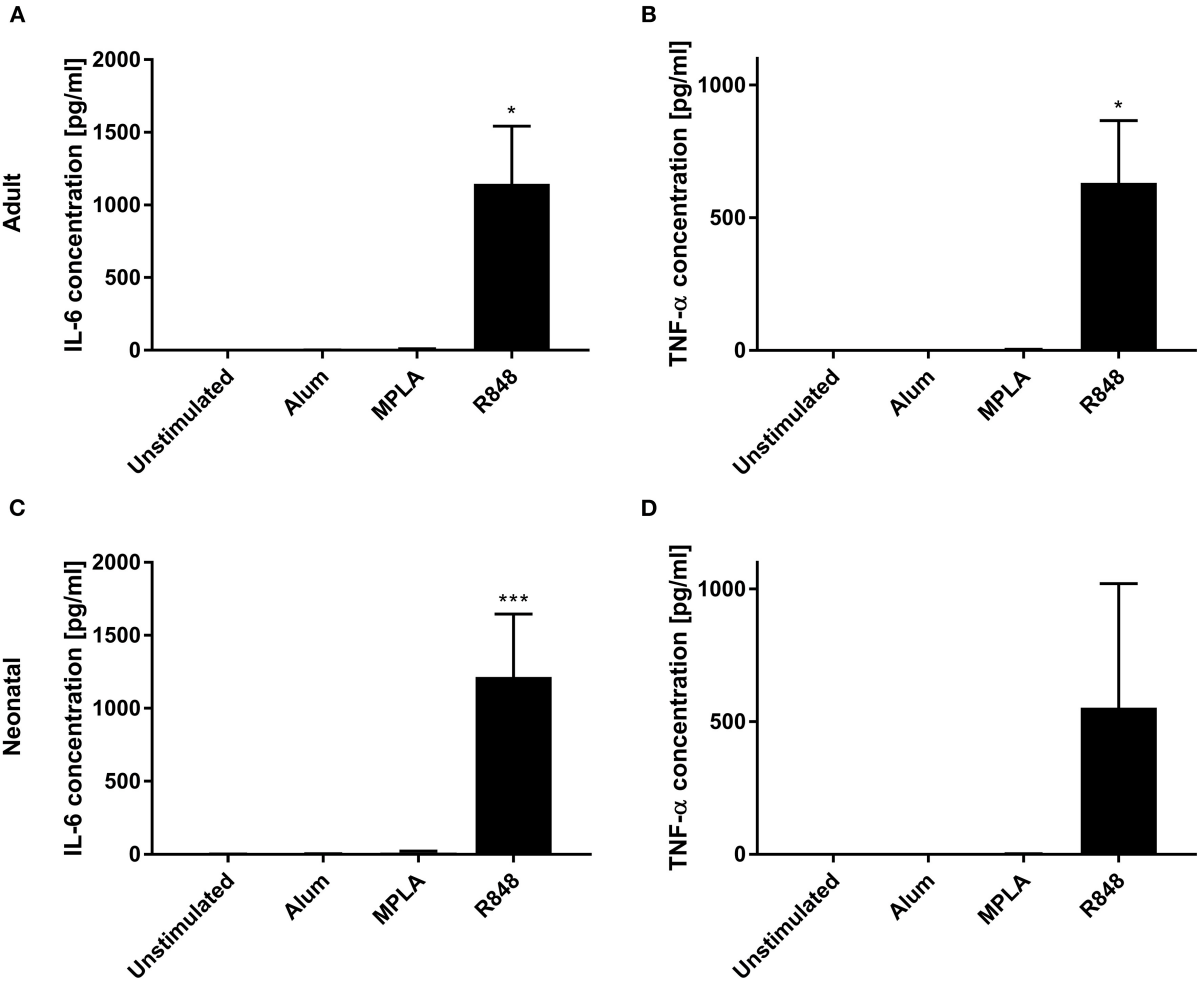
Effect of different adjuvants on DC cytokine secretion. Analysis of IL-6 **(A,C)** and TNF-α **(B,D)** secretion by adult **(A,B)** and neonatal **(C,D)** DCs after incubation with or without alum, MPLA or R848 for 20 h. Data represent the means ± SD (*n* = 3). Significantly more than unstimulated: *P < 0.05; ***P < 0.005 [**(A,B)** one-way ANOVA; **(C,D)** Kruskal-Wallis test].

Three adjuvants: Alum, MPLA, and R848 were analyzed for their immune-stimulatory activities by incubating cDC2 and pDCs together with or without adjuvant. The number of CD1c^+^CD80^+^ double-positive cells increased from 9% to 71% after stimulation with R848, whereas alum and MPLA only exerted marginal effects, i.e., 19% and 15% positive cells, respectively (Figure 1A). Additionally, stimulation with R848 increased the expression of CD83 from 16 to 59% on adult cDC2 (Figure 1B). Similar trends were observed after the incubation of neonatal DCs with R848 (Figures 1C,D). R848 stimulation also led to a substantial upregulation of CD80 expression on adult and neonatal pDCs, i.e., the number positive cells increased by 40–50%, whereas stimulation with alum or MPLA had only minor effects (Figures 2A,C). The upregulation of CD83 after R848 stimulation was not as pronounced but the amount of CD123^+^CD83^+^ double-positive cells still increased two-fold on adult and 3.3-fold on neonatal cells (Figures 2B,D). In addition to increased activation marker expression, R848 stimulated a substantiate secretion of IL-6 and TNF-α in both adult and neonatal DCs. Alum and MPLA, however, failed to induce any cytokine production (Figure 3).

### PEGylation of R848 does not abrogate its stimulatory activity

**Figure 4:**
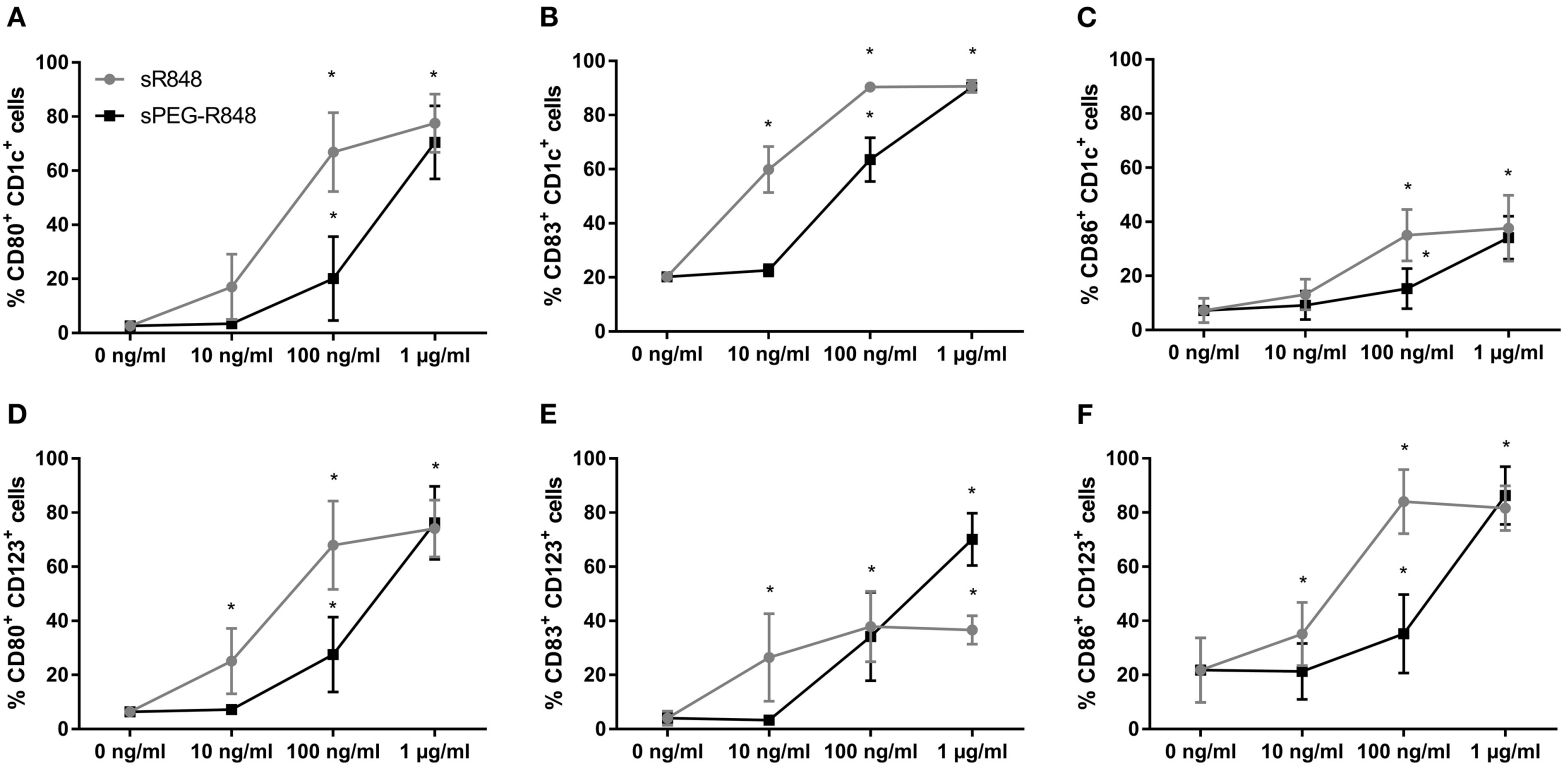
R848 retains stimulatory activity after PEGylation. Flow cytometric analysis of adult CD1c^+^ **(A–C)** and CD123^+^ **(D–F)** DCs after stimulation with different concentrations of R848 and PEG-R848 for 20h. CD80- **(A,D)**, CD83- **(B,E)** and CD86-expressing **(C,F)** cells were evaluated. Data represent the means ± SD (*n* = 3). Significantly greater than cells incubated with 0 ng/ml: *P < 0.05 (two-way ANOVA).

To enable the quantification of R848 in OVA NCs and prevent diffusion after encapsulation, R848 was conjugated to a polyethylene glycol (PEG) chain (24). The impact of PEGylation on PEG-R848 was evaluated at multiple concentrations (Figure 4). DCs incubated with lower concentrations of PEG-R848 exhibited an approximately ten-fold decrease in activation relative to cells incubated with the same concentration of R848. Especially at the lowest concentration (10 ng/ml), neither CD1c^+^ nor CD123^+^ cells showed any increase in activation relative to the negative control. At the highest concentration tested (1 μg/ml), however, there was no detectable difference between PEG-R848 and R848. Coupling PEG-R848 to Rhodamine B (RhB) made it possible to directly correlate activation marker expression and the internalization of PEG-R848 by cells (Supplementary Figure S2). At 10 ng/ml, neither CD1c^+^ nor CD123^+^ RhB^+^ cells were measurable. At 100 ng/ml the number increased to 13.3% and 54.8%, respectively. At 1 μg/ml, nearly all cells were RhB^+^.

### OVA-PEG-R848 NCs serve as an adjuvant-antigen co-delivery system for DC activation

Co-delivery of soluble adjuvants and antigens is plagued by their rapid dissociation (25). To circumvent this issue, PEG-R848 was encapsulated in OVA NCs (OVA-PEG-R848 NCs), and the capacities of PEG-R848 and OVA-PEG-R848 NCs to activate DCs were assessed and compared. The average size of OVA NCs was 240 nm in toluene and 160 nm in water with a zeta potential of −26.4 (Supplementary Table S1). The observed encapsulation efficiency of OVA-PEG-R848 NCs was approximately 70% (± 5%) of payload retained in NCs, similar to our previous reported data (Supplementary Figure S3A) (24). Moreover, OVA NCs did not show signs of payload leakage after transferring them to water and washing away any non-encapsulated molecules (Supplementary Figure S3B).

**Figure 5:**
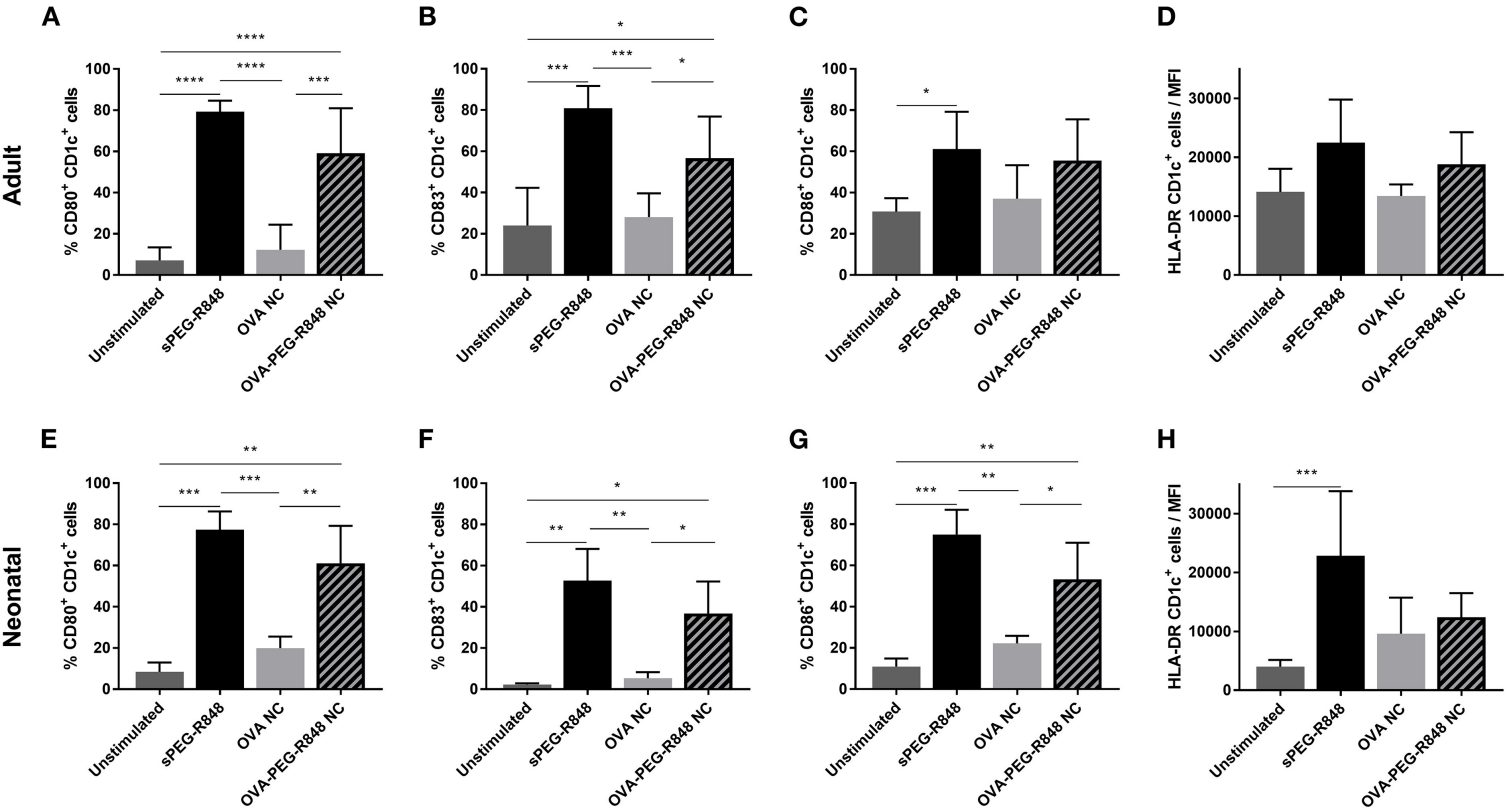
Stimulation of CD1c^+^ cDC2 by OVA-PEG-R848 NCs. Flow cytometric analysis of adult **(A–D)** and neonatal **(E–H)** CD1c^+^ DCs after incubation with soluble PEG-R848, empty OVA NCs or OVA-PEG-R848 NCs. Frequency of CD80^+^ **(A,E)**, CD83^+^ **(B,F)** and CD86^+^ **(C,G)** cells as well as HLA-DR mean fluorescent intensity **(D,H)** was evaluated. Data represent the means ± SD [**(A–D)**, n = 5; **(E–H)**, n = 3). Significantly different: *P < 0.05; **P < 0.01; ***P < 0.005; ****P < 0.0001 (one-way ANOVA).

DCs were incubated with OVA-PEG-R848 NCs (effective 500 ng/ml R848 concentration), empty OVA NCs or soluble PEG-R848 in equimolar concentrations. Both adult and neonatal CD1c^+^ cells were significantly activated by incubation with OVA-PEG-R848 NCs (Figure 5). After stimulation, 59% of adult and 61% of neonatal DCs expressed cell surface CD80, compared to 7–8% for the negative control (Figures 5A,E). CD83 expression by both adult and neonatal DCs also increased more than two-fold (Figures 5B,F). Cells incubated with empty NCs, in contrast, showed only a negligible increase in marker expression, clearly demonstrating adjuvant-specific DC activation.

**Figure 6:**
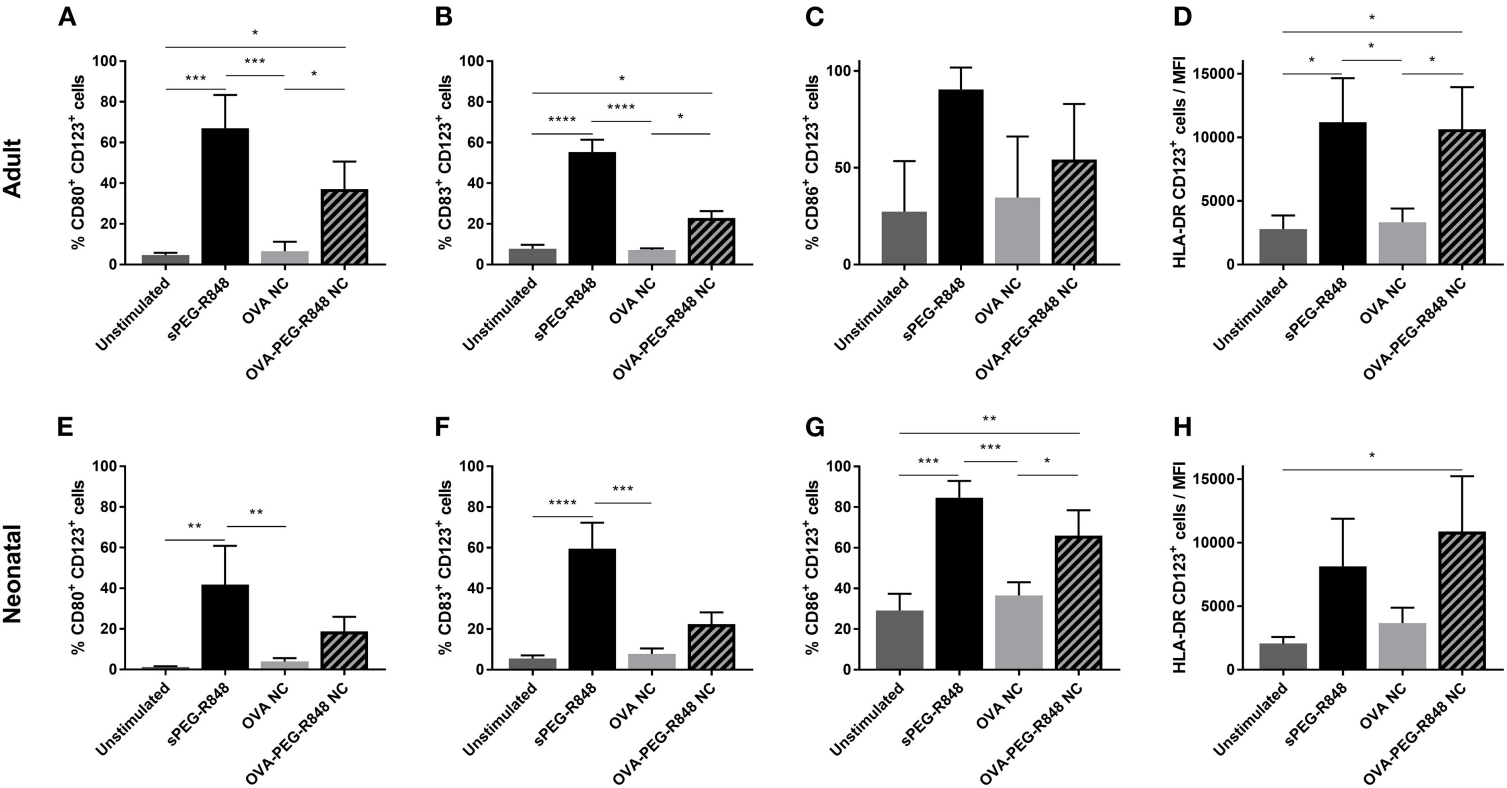
Stimulation of CD123^+^ pDCs by OVA-PEG-R848 NCs. Flow cytometric analysis of adult **(A–D)** and neonatal **(E–H)** CD123^+^ DCs after incubation with soluble PEG-R848, empty OVA NCs or OVA-PEG-R848 NCs. CD80^+^ **(A,E)**, CD83^+^ **(B,F)** and CD86^+^ **(C,G)** cells, and HLA-DR mean fluorescent intensity **(D,H)** were evaluated. Data represent the means ± SD (*n* = 3). Significantly different: *P < 0.05; **P < 0.01; ***P < 0.005; ****P < 0.0001 (one-way ANOVA).

Activation of CD123^+^ cells was not as pronounced as for CD1c^+^ DCs, but a significant increase in activated cells could still be measured (Figure 6). The expression of CD80 increased from 5 to 37% in adult and 1% to 19% in neonatal DCs (Figures 6A,E). Similarly, the number of CD83^+^ cells increased from 5–8% to 22% for both adult and neonatal cells (Figures 6B,F). Strikingly, the HLA-DR mean-fluorescent intensity (MFI) increased substantially more in the CD123^+^ (Figures 6D,H), than the CD1c^+^, population (Figures 5D,H).

For all DC populations and markers tested, soluble PEG-R848 treatment resulted in a higher activation than treatment with OVA-PEG-R848 NCs. This was principally due to significantly greater uptake of soluble PEG-R848 (Supplementary Figure S4). Only 54–64% of CD1c^+^ DCs were RhB^+^ after incubation with OVA-PEG-R848 NCs compared to 98% of cells incubated with soluble PEG-R848 (Supplementary Figures S4A,C). The difference was even more pronounced when assessing uptake by CD123^+^ cells exhibiting a >70% reduction in RhB^+^ cells (Supplementary Figures S4B,D). After normalizing the expression of CD80 and CD83 in terms of RhB^+^ levels of OVA-PEG-R848 stimulated cells, and therefore to the amount of ingested PEG-R848, it was evident that stimulation with OVA-PEG-R848 led to slightly higher up-regulation compared to equal amounts of ingested, soluble adjuvant for both the CD1c^+^ and CD123^+^ DC populations (Supplementary Figure S5).

**Figure 7:**
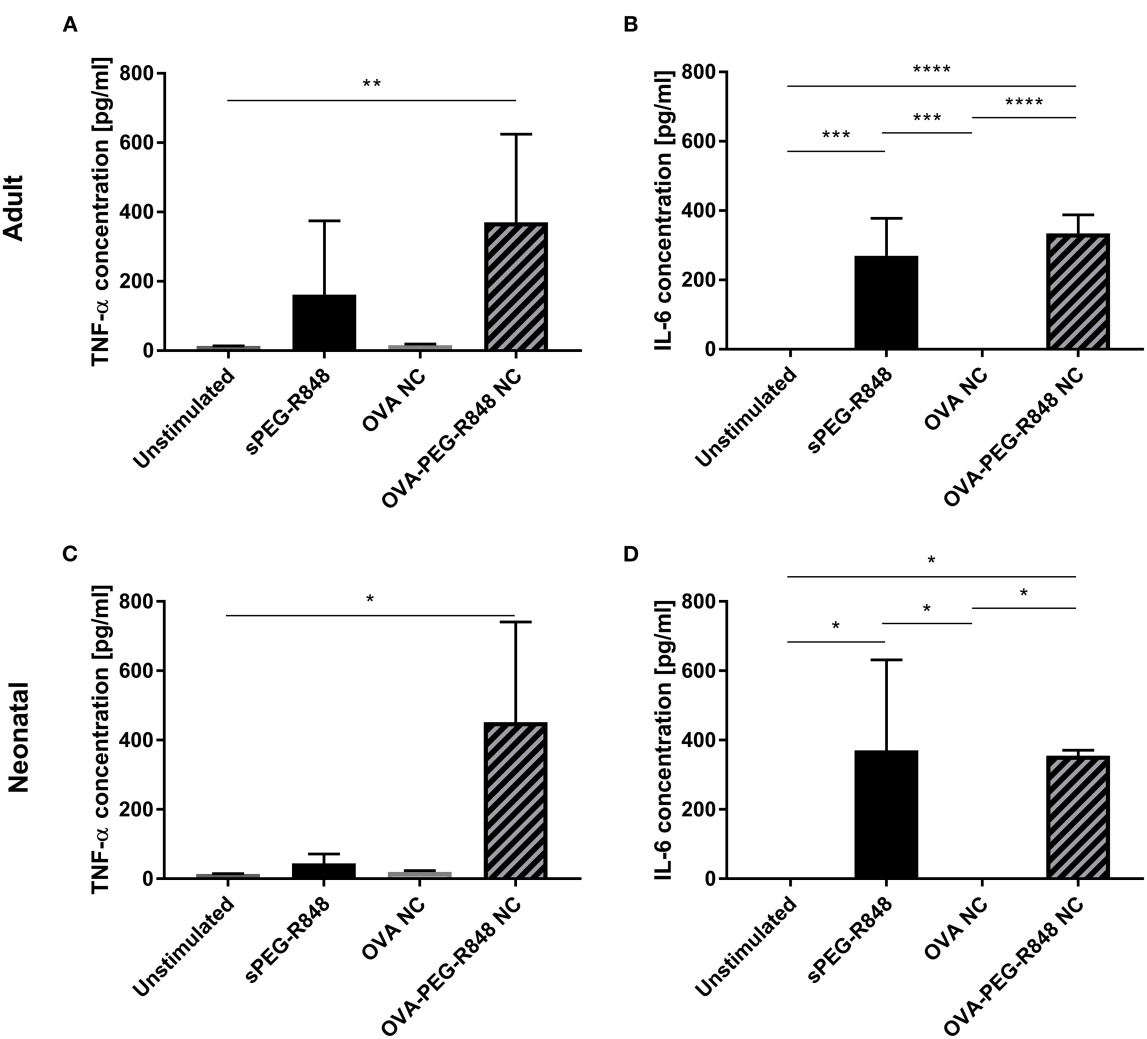
PEG-R848 encapsulation increases cytokine secretion. Analysis of TNF-α **(A,C)** and IL-6 **(B,D)** secretion by adult **(A,B)** and neonatal **(C,D)** DCs after incubation with soluble PEG-R848, empty OVA NCs or OVA-PEG-R848 NCs. Data represent the means ± SD (*n* = 3). Significantly different: *P < 0.05; **P < 0.01; ***P < 0.005; ****P < 0.0001 (one-way ANOVA).

Analysis of cell culture supernates showed that OVA-PEG-R848 NCs induced an increase in IL-6 and, especially, TNF-α production (Figure 7). Adult and neonatal cells incubated with OVA-PEG-R848 NCs secreted even more TNF-α than cells incubated with soluble PEG-R848 (Figures 7A,C). Additionally, IL-6 secretion by adult cells was slightly increased compared to the positive control (Figure 7B).

## Discussion

In 2019, infectious diseases accounted for roughly half of the deaths in children under the age of five; pneumonia and diarrhea alone accounted for 23% of total deaths (1). Many of these cases could be preventable if vaccines, administered during the first weeks of life, elicited a more robust immune response. Most routine vaccines are administered in a three-dose regimen at 2, 4 and 6 months after birth to achieve maximum protection. This, however, leaves children vulnerable to infectious diseases in the first 6 months of life (26). The development and administration of more potent adjuvants could increase vaccine immunogenicity and infant protection. R848, the focus of this study, is a TLR7/8 agonist, which is currently approved for topical use to treat skin lesions and cutaneous T cell lymphoma (27, 28).

Initial experiments showed that R848 was a much more potent adjuvant than either alum or MPLA. Noteworthy, so far most of the studies reporting stimulatory capacities of MPLA and alum were performed in artificially generated DCs (moDCs) (29–31). In this context, primary DCs represent more realistic functional characteristics for the evaluation and screening of potential adjuvants for vaccine development, especially in pediatrics. In this regard, adult primary DCs stimulated with R848 exhibited significantly greater expression of co-stimulatory receptors, CD80 and CD83, and increased secretion of pro-inflammatory cytokines, IL-6 and TNF-α. R848 also activated neonatal DCs isolated from cord blood, confirming the reports of other groups (32, 33).

The rapid dissociation of small molecules co-delivered with antigen upon injection is a major issue (25). Moreover, systemic administration of R848 alone is linked to side effects (34, 35). Encapsulation of R848 in full-protein NCs solves both issues simultaneously. Previously, OVA NCs with three varying degrees of crosslinking were compared for their abilities to release their cargo and subsequently activate adult CD1c^+^ DCs. It was shown that NC crosslinking density directly influences the release of cargo and that only low-crosslinked NCs successfully activate primary DCs. PEGylation of R848, however, was crucial to avoid diffusion of the small molecule out of low-crosslinked NCs (24), In addition, conjugating RhB to PEG-R848 made it possible to precisely define the amount of encapsulated R848 inside OVA NCs and to measure the number of adjuvant-ingesting cells. PEGylation reduced the effect of R848 by approximately ten-fold when used at lower concentrations to stimulate both CD1c^+^ and CD123^+^ DC populations. This difference was no longer observed at the highest concentration tested, a finding that correlated directly with the percentage of RhB^+^ DCs. OVA-PEG-R848 NCs activated both adult and neonatal primary DCs. CD80, CD83 and CD86 expression levels were elevated significantly following NC stimulation. Notably, incubation with empty OVA NCs did not affect marker expression, confirming the adjuvant-specific effect. Furthermore, OVA-PEG-R848 NCs treatment stimulated the secretion of pro-inflammatory cytokines, i.e., IL-6 and TNF-α, markedly.

It is worth to mention that the study presented here, together with our previous studies regarding this nanosystem (24), provide data supporting the consequent evaluation and characterization of this nanoformulation *in vivo*. While this study yields answers to relevant immunological questions, such as the possibility to elicit a robust immune stimulation of both primary adult and neonatal DCs, consecutive *in vivo* studies would need to be performed to provide a complete pre-clinical overview of this nanovaccine platform.

Previous *in vitro* and *in vivo* studies showed similar results involving R848 encapsulated in different carrier systems (36–38). In a neonatal context Dowling *et al*. were able to show the potential of TLR7/8 agonists by encapsulating the agonist CL075. Stimulation of monocyte-derived dendritic cells (moDCs) with CL075 nanoparticles upregulated costimulatory markers and led to an increased secretion of pro-inflammatory cytokines (39). The present study, however, is one of the first to evaluate the stimulatory effects of encapsulated R848 on neonatal primary DCs, providing further proof that R848 is a more potent adjuvant, relative to alum or MPLA, to incorporate into neonatal vaccines. While moDCs and cDCs share a lot of similarities, e.g., the upregulation of costimulatory markers and secretion of IL12p70 upon activation, cDCs overall possess higher migratory as well as T cell activating capabilities (40–42). Further evidence regarding the potential of R848 was also provided in recent studies investigating the effect of conjugating R848 to inactivated influenza virus. Non-human primates vaccinated with conjugated virus showed an increase in activated B cells, DCs and macrophages, as well as elevated antibody titers still measurable 6 months after vaccination (43–45).

HBV vaccine, requiring the administration of three doses over a 6-month period to achieve full protection, could benefit immensely from the increased stimulatory activity of R848 (16). Vaccinating infants three times can be challenging especially in low-income countries where HBV tends to be most prevalent (19, 46). Licensed HBV vaccines are currently adjuvanted with aluminum-based agents (47, 48). Unfortunately, aluminum salts only elicit a T_H_2-type immune response, however neonatal immunity is already naturally skewed toward this direction (5, 49, 50). Therefore, alum-based vaccines do not sufficiently activate the neonatal immune system nor do they generate specific CD4^+^ and CD8^+^ T cells needed to prevent chronic infections (51, 52). This is a significant issue insofar as 90% of HBV infections that occur following mother-to-infant transmission develop into chronic disease (53). It is relevant to note, therefore, that nanocapsules composed of HBV surface antigen (HBsAg) and functionalized with MPLA bear the capacity to generate a specific T cell response *in vitro* (54).

In summary, R848 is a potent adjuvant capable of activating adult and neonatal primary DCs *in vitro*. PEGylation of R848 only exerted a minor impact on its effect; labeling with RhB made it possible to track the number of adjuvant-ingesting cells and quantify the amount of R848 inside NCs. Stimulation with functionalized OVA NCs led to a marked increase in the expression of activation markers and secretion of pro-inflammatory cytokines by conventional and plasmacytoid DCs. This study is among the first to demonstrate the capacity of encapsulated R848 to activate neonatal DCs. This finding supports the potential incorporation of R848 as adjuvant in neonatal vaccines, making them more effective in eliciting a robust immune response.

## Data availability statement

The datasets generated and/or analysed during the current study are available from the corresponding author on reasonable request.

## Ethics statement

The studies involving human participants were reviewed and approved by Landesärztekammer Rheinland-Pfalz. Written informed consent to participate in this study was provided by the participants' legal guardian/next of kin.

## Author contributions

SW, MM, MF, MC, HT-A, KL, and SG contributed to the study conception and design. Material preparation and data collection were performed by SW, MM, FB, LP, and MF. Data analysis was performed by SW and MF. The first draft of the manuscript was written by SW and all authors commented on previous versions of the manuscript. All authors read and approved the final manuscript.

## Funding

This project was funded by the Else Kröner-Fresenius Stiftung (EKFS) grant 2016_A206 and by the Deutsche Forschungsgemeinschaft (DFG, German Research Foundation)—Project-ID 213555243—SFB1066.

## Conflict of interest

The authors declare that the research was conducted in the absence of any commercial or financial relationships that could be construed as a potential conflict of interest.

## Publisher's note

All claims expressed in this article are solely those of the authors and do not necessarily represent those of their affiliated organizations, or those of the publisher, the editors and the reviewers. Any product that may be evaluated in this article, or claim that may be made by its manufacturer, is not guaranteed or endorsed by the publisher.
