# Supplementary material for: OVA-PEG-R848 nanocapsules stimulate neonatal conventional and plasmacytoid dendritic cells
Source: Front Pediatr. 2022 Sep 13;10:966113. doi: 10.3389/fped.2022.966113 (PMC9513203; doi:10.3389/fped.2022.966113)
Supplement: Supplementary file 1 [file Data_Sheet_1.docx]

**Low-crosslinked OVA-PEG-R848 Nanocapsules Stimulate Neonatal Conventional and Plasmacytoid Dendritic Cells**

Sebastian Wirsching^1^, Marina Machtakova^3^, Frauke Borgans^1,4^, Leah Pretsch^1^, Michael Fichter^1,2,3^, Maximiliano Luis Cacicedo^1^, Heloise Thérien-Aubin^3,5^, Katharina Landfester^3^, Stephan Gehring^1*^

^1^Children’s Hospital, University Medical Center of the Johannes Gutenberg University Mainz, Langenbeckstr. 1, 55131 Mainz, Germany

^2^Department of Dermatology, University Medical Center of the Johannes Gutenberg University Mainz, Langenbeckstr. 1, 55131 Mainz, Germany

^3^Max Planck Institute for Polymer Research, Ackermannweg 10, 55128 Mainz, Germany

^4^Department of Infectious Diseases, University Hospital Frankfurt, Theodor-Stern-Kai 7, 60590 Frankfurt, Germany

^5^Department of Chemistry, Memorial University of Newfoundland, 1 Artic Ave, A1B 3X7, St. John’s, Canada

Address correspondence to:

Prof. Dr. med. Stephan Gehring

Children's Hospital, University Medical Center, Johannes Gutenberg University

Langenbeckstraße 1

55131 Mainz, Germany

Electronic address: stephan.gehring@uni-mainz.de

Telephone: +49 (0) 6131 17-3560

**Supplemental Material**

**Table 1: Physiochemical characterization of OVA NCs**

|  | **Toluene** |  | **Water** |  |  |
| --- | --- | --- | --- | --- | --- |
|  | **Diameter [nm]** | **PDI** | **Diameter [nm]** | **PDI** | **ζ-Potential [mV]** |
| **OVA NC** | **240** | **0.22** | **160** | **0.13** | **-21.8** |

**
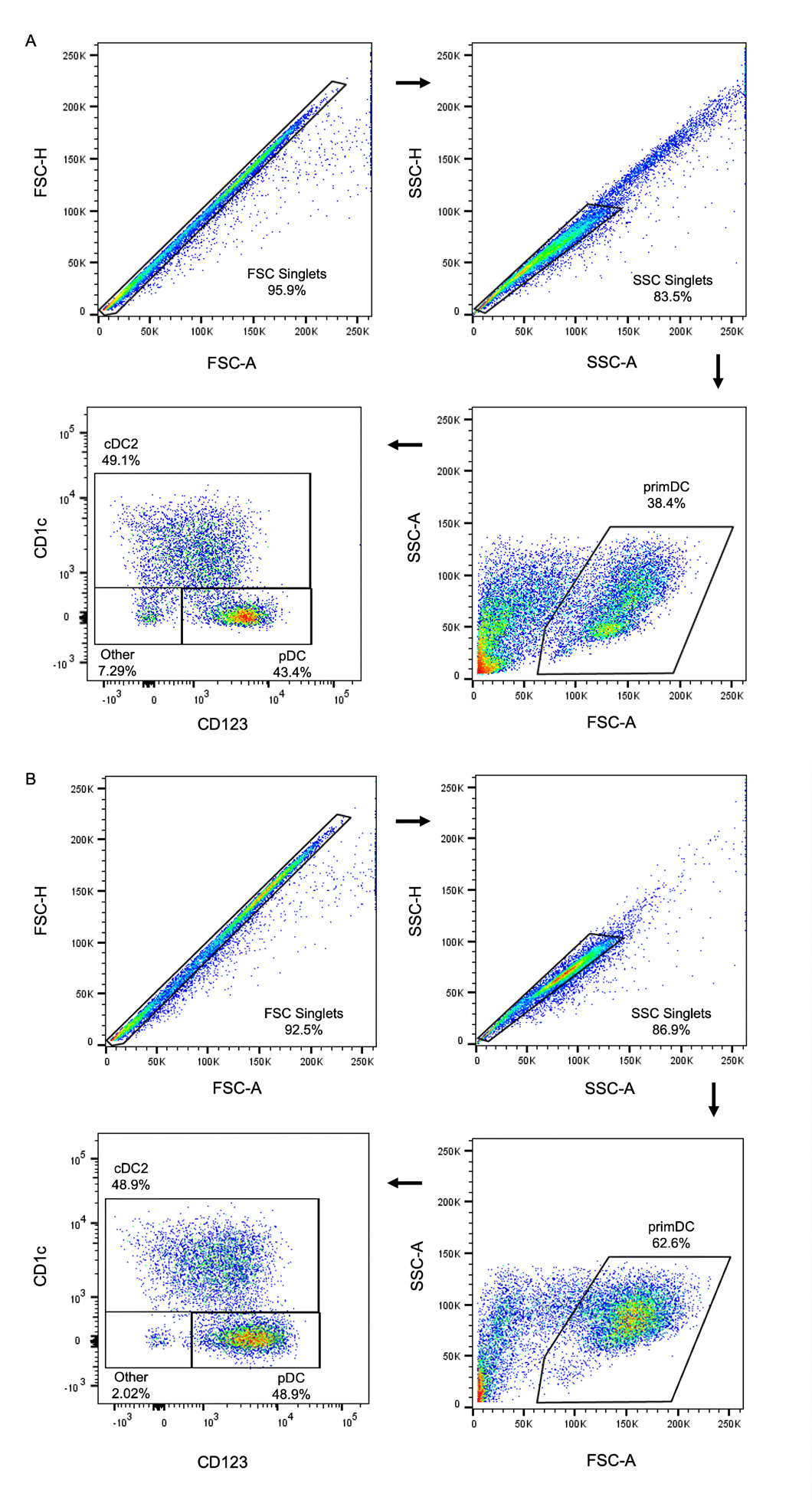
**

**Figure S1: cDC and pDC gating strategy.** A) Gating of unstimulated primary DCs after 24h culture. B) Gating of primary DCs after culturing them with PEG-R848 [1 µg/ml] for 24h.

**
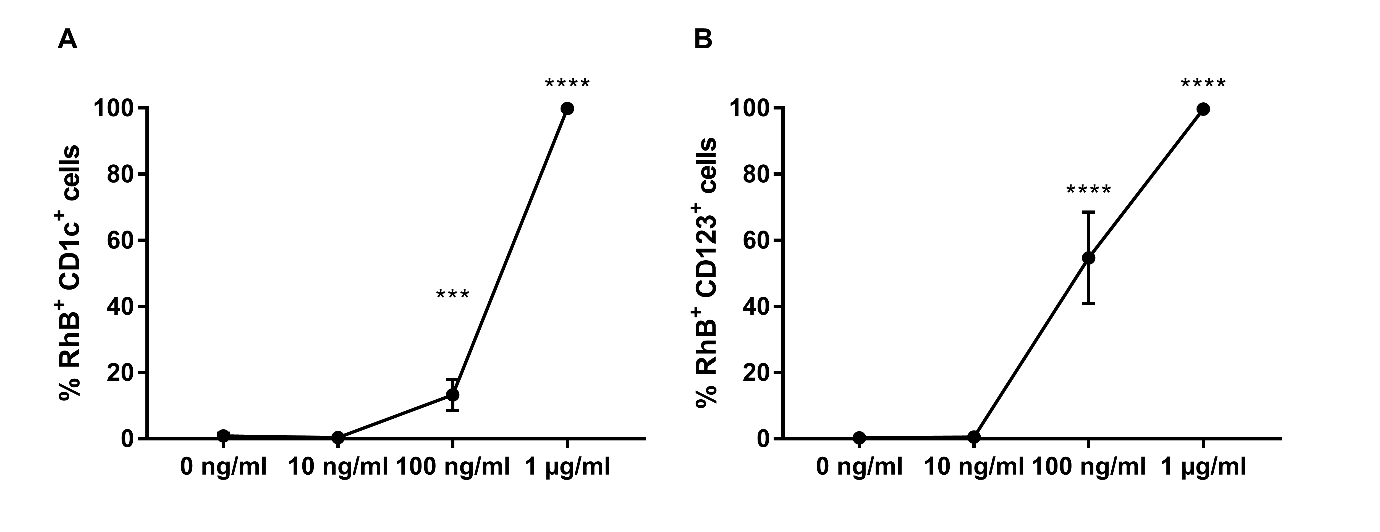
****Figure S2:** **Uptake of soluble PEG-R848.** Flow cytometric analysis of DCs after incubation with soluble PEG-R848, empty OVA NCs or OVA-PEG-R848 NCs. Percentage of RhB^+^ CD1c^+^ (A) and CD123^+^ (B) cells. Data represent the means ± SD (n = 3). Significantly greater than 0 ng/ml control: ****P*< 0.005; *****P*< 0.001 (one-way ANOVA).

**
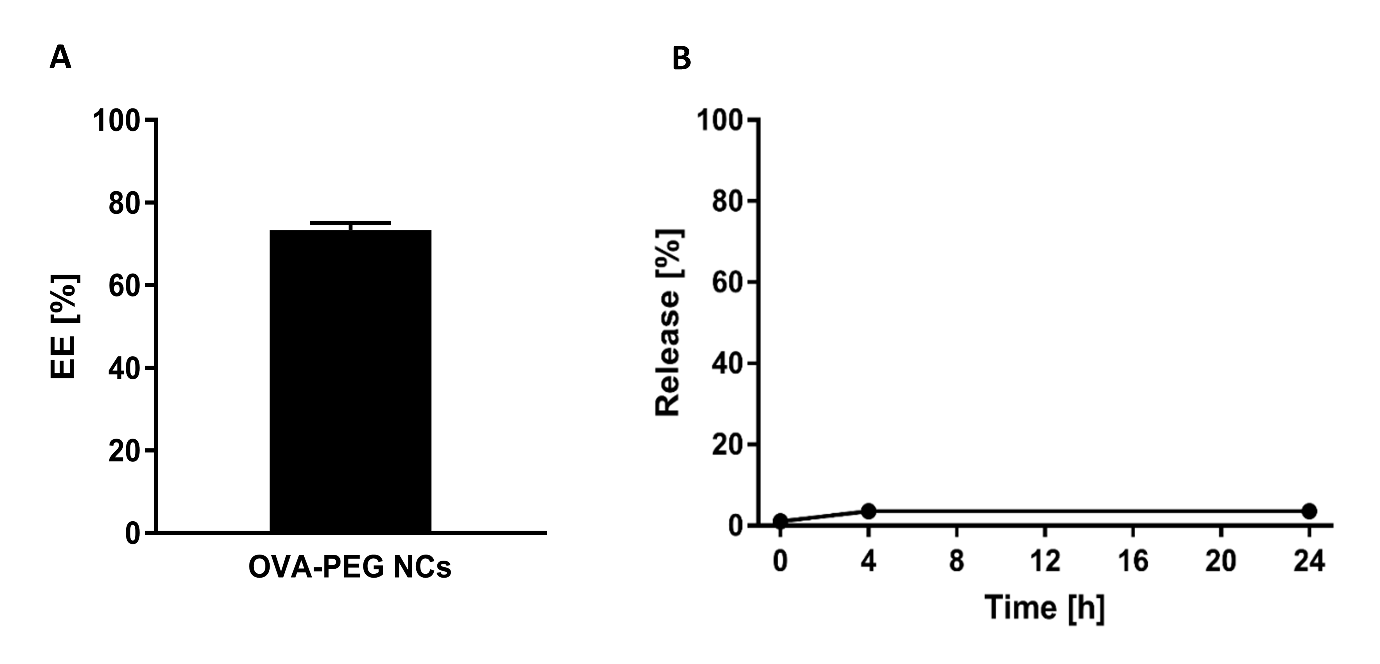
**

**Figure S3: Encapsulation and release kinetics of OVA-PEG NCs.** A) Encapsulation efficiency of PEG-Rhodamine in OVA NCs. B) Release of PEG-Rhodamine from OVA NCs after 0, 4 and 24 hours.

**
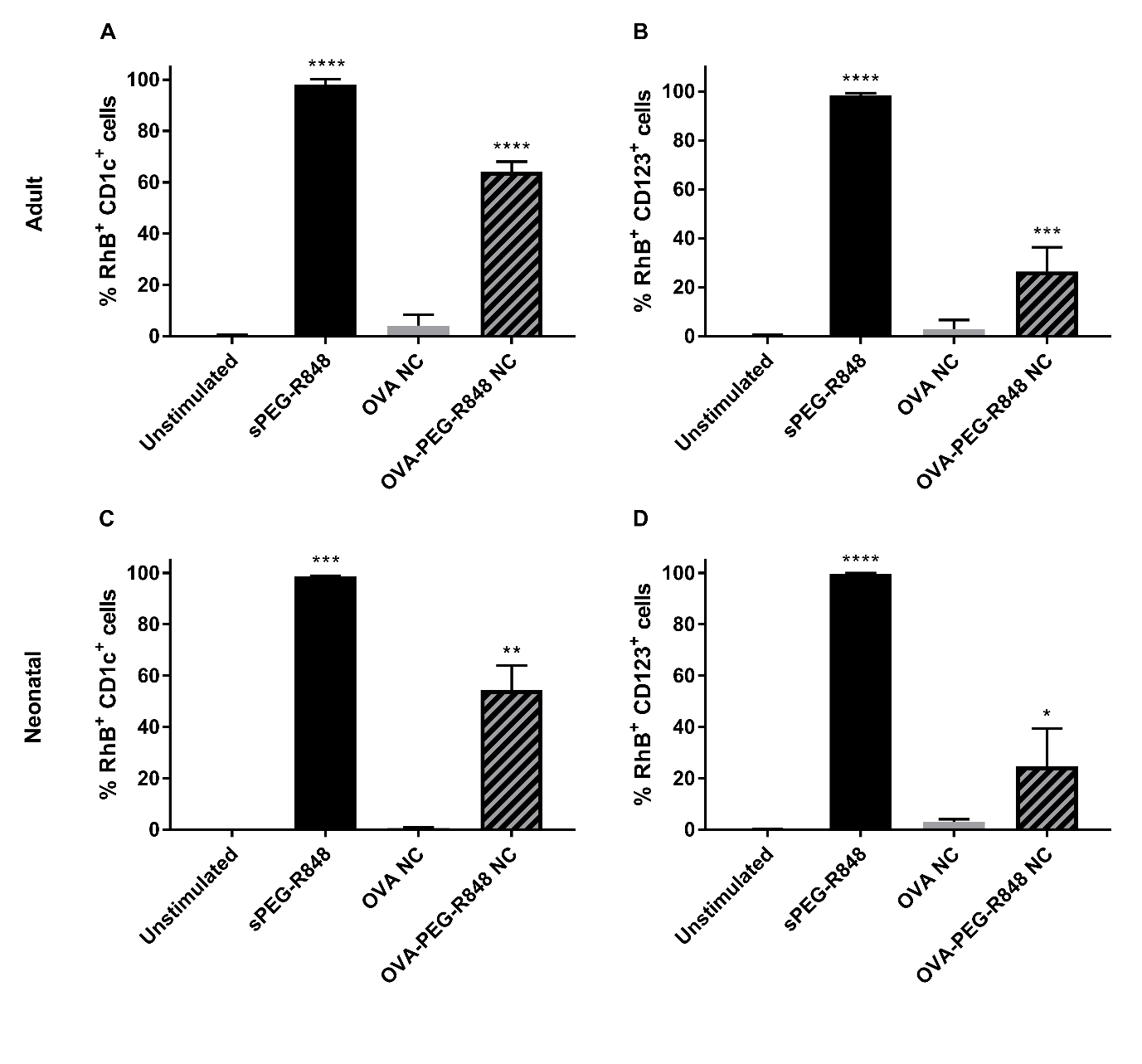
**

**Figure S4: Uptake of soluble PEG-R848 and OVA-PEG-R848 NCs.** Flow cytometric analysis of adult (A, B) and neonatal (C, D) DCs after incubation with soluble PEG-R848, empty OVA NCs or OVA-PEG-R848 NCs. Percentage of RhB^+^ CD1c^+^ (A, C) and CD123^+^ (B, D) DCs. Data represent the means ± SD (n = 3). Significantly greater than unstimulated: ***P*< 0.001; ****P*< 0.005 (one-way ANOVA).

**
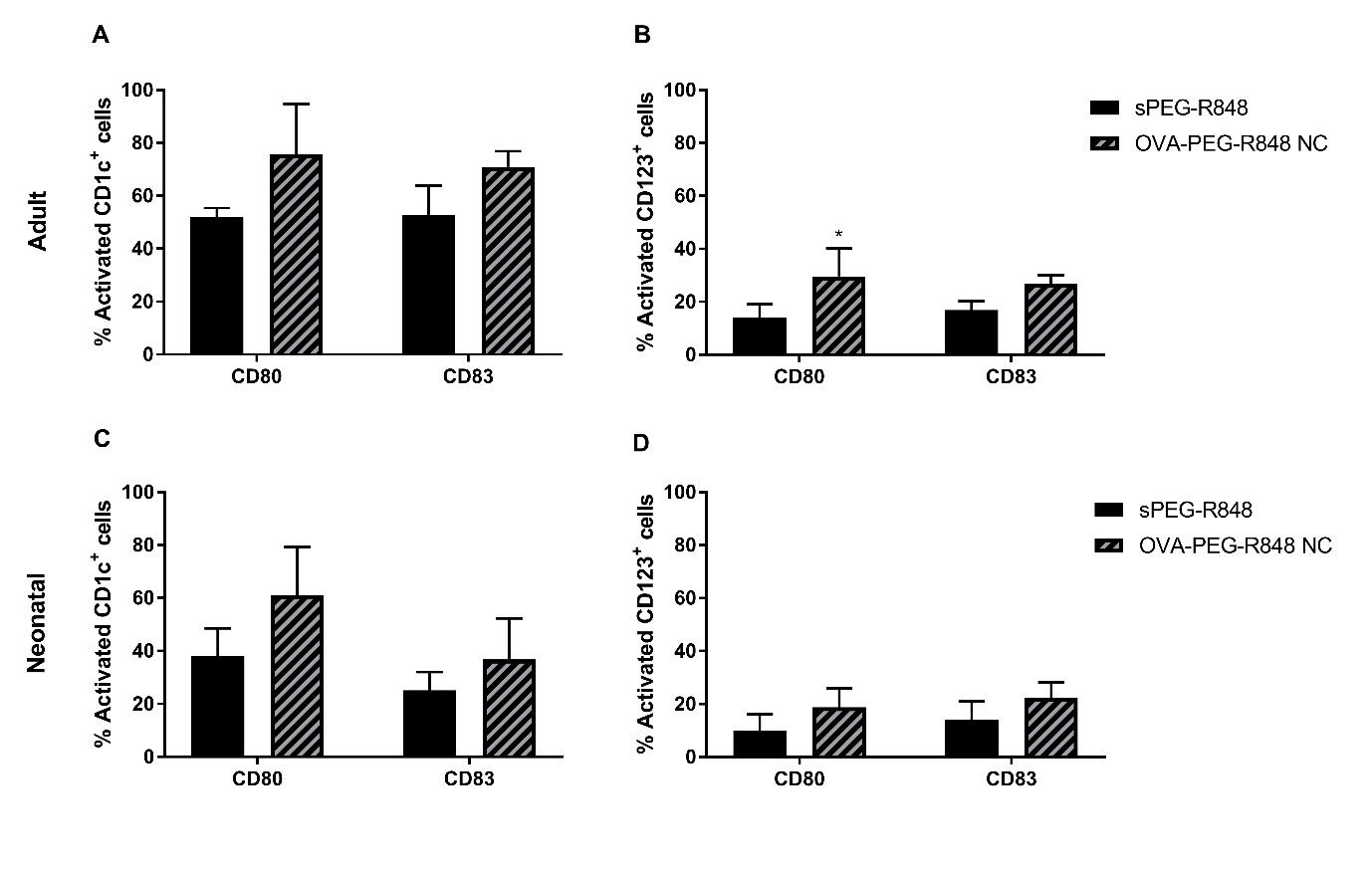
**

**Figure S5: DCs are activated more effectively by OVA-PEG-R848 than by soluble PEG-R848.** Flow cytometric analysis of adult (A, B) and neonatal (C, D) DCs after incubation with soluble PEG-R848, empty OVA NCs or OVA-PEG-R848 NCs. Percentage of CD80^+^ or CD83^+^ CD1c^+^ (A, C) and CD123^+^ (C, D) DCs. Activation data were normalized to the percentage of RhB^+^ cells after incubation with OVA-PEG-R848. Data represent the means ± SD (n = 3).
